# The fraction of lung cancer attributable to smoking in the Norwegian Women and Cancer (NOWAC) Study

**DOI:** 10.1038/s41416-020-01131-w

**Published:** 2020-10-27

**Authors:** Merethe S. Hansen, Idlir Licaj, Tonje Braaten, Eiliv Lund, Inger Torhild Gram

**Affiliations:** Faculty of Health Sciences, Department of Community Medicine, The UiT Arctic University of Norway, Tromsø, Norway

**Keywords:** Epidemiology, Oncology

## Abstract

**Background:**

We examined the association between active and passive smoking and lung cancer risk and the population attributable fraction (PAF) of lung cancer due to active smoking, in the Norwegian Women and Cancer Study, a nationally representative prospective cohort study.

**Methods:**

We followed 142,508 women, aged 31–70 years, who completed a baseline questionnaire between 1991 and 2007, through linkages to national registries through December 2015. We used Cox proportional hazards models, to estimate hazard ratios (HRs) with 95% confidence intervals (CIs). We calculated PAF to indicate what proportion of lung cancer cases could have been prevented in the absence of smoking.

**Results:**

During the more than 2.3 million person-years of observation, we ascertained 1507 lung cancer cases. Compared with never smokers, current (HR 13.88, 95% CI 10.18–18.91) smokers had significantly increased risk of lung cancer. Female never smokers exposed to passive smoking had a 1.3-fold (HR 1.34, 95% CI 0.89–2.01) non- significantly increased risk of lung cancer, compared with never smokers. The PAF of lung cancer was 85.3% (95% CI 80.0–89.2).

**Conclusion:**

More than 8 in 10 lung cancer cases could have been avoided in Norway, if the women did not smoke.

## Background

Lung cancer is the third most common cancer in women worldwide.^1^ Active smoking is the main cause of lung cancer. The incidence of lung cancer among women in Western Europe has plateaued in recent years.^2^ In Norway, the incidence rate for lung cancer in women decreased in 2017. If this reduction continues, 2015 will be the top year with the highest incidence of lung cancer among Norwegian women, with a rate of 53.1 per 100,000.^3^ In 2017, the incidence rate of lung cancer among Norwegian women were 52.0 per 100,000.^4^ The incidence trend of lung cancer among Norwegian women is similar to that in Denmark, but steeper than those observed in Sweden and Finland.^5^

Among Norwegian women, the smoking prevalence increased sharply from 1920 and towards 1950 when 20% of women were smokers.^6^ The prevalence of daily smoking in Norwegian women peaked at 37% around 1970.^6^ Today, 12% of Norwegian women are daily smokers.^7^ However, lung cancer is also a significant health problem among those exposed to passive smoking.^8,9^ In Northern Europe, approximately 26% of lung cancer cases occur in women who have never smoked.^10^ The IARC Monograph from 2004, The US Surgeon General and the latest World Cancer Report state that exposure to involuntary smoking increases lung cancer risk by ~20–30%.^1,11,12^ Other known risk factors for lung cancer are exposure to asbestos, radon, polycyclic aromatic hydrocarbons, and emissions from household combustion of coal.^2^

Few prospective cohort studies have been able to study both active and passive smoking, and the lung cancer risk they confer among women.

We utilised the Norwegian Women and Cancer Study, a nationally representative prospective cohort, to estimate the risk of lung cancer associated with active and passive smoking. Furthermore, we estimated the number of lung cancer cases that could have been avoided in the absence of smoking in Norway in 2015.

## Methods

### Study population

The Norwegian Women and Cancer Study cohort profile has been previously described in detail.^13^ The NOWAC Study is initiated from the University of Tromsø–The Arctic University of Norway. Briefly, the Central Population Register selected a random sample of women according to the year of birth. Subsequently, an invitation to participate in the study together with a baseline questionnaire and a pre-stamped return envelope enclosed was mailed to each woman. All women gave informed consent (https://site.uit.no/nowac/). The National Data Inspectorate and the Regional Committee for Medical Research Ethics approved the study.

Women who completed a questionnaire during three waves of data collection: 1991–1992, 1996–1997, and 2003–2007 (172,478), were included. The overall response rate was 52.7%. We excluded women with prevalent cancer (*n* = 6664), those who emigrated (*n* = 64), or died before the start of follow-up (*n* = 10), those with an age at the exit that was below age at recruitment (*n* = 32), those with missing information on smoking status (*n* = 590), and never smokers with missing information on passive smoking (*n* = 10,879). Finally, we excluded those with missing information on the covariates education and alcohol consumption (*n* = 11,731). Altogether, 349 women with lung cancer were excluded in this process. The cohort comprised 142,508 women and 1507 lung cancer cases.

### Exposure information

As described previously^14^ the questionnaire included a detailed assessment of smoking habits, if their parents smoked during childhood, if they lived with a smoker as adults, physical activity, alcohol consumption as well as the height and current weight (which were used to compute body mass index (BMI) as weight in kilograms divided by the square of height in metres). The questionnaires asked if the women had ever been smoking, and those answering ‘yes’ were asked for the number of cigarettes smoked daily at different age intervals. Subsequently, they were asked if they smoked on a daily basis at present. We categorised ever smokers according to current and former smoking status, age at smoking initiation, smoking duration, the average number of cigarettes smoked daily, pack-years of smoking (i.e., number of cigarettes smoked per day, divided by 20, multiplied by the number of years smoked), all at enrolment. Former smokers were classified according to years since quitting smoking. All women who were neither current nor former smokers were classified as never smokers. Among never smokers, those who reported that their parents smoked during childhood or they lived with a smoker as adults were classified as passive smokers. We calculated average alcohol consumption in g/day among drinkers based on the content of pure alcohol in different beverages and usual portion sizes in Norway.

### Follow up

As previously described^15^ we followed the women through linkages to the Cancer Registry of Norway and the Norwegian Central Population Register to identify all cancer cases, and emigrations and deaths, respectively, using the unique 11-digit national personal identification number. We calculated person-years from the start of follow-up to any incident cancer diagnosis (except basal cell carcinoma), emigration, death or the end of follow-up (31 December 2015), whichever came first. We classified lung cancer cases according to the original codes in the International Classification of Diseases, Seventh Revision.

### Statistical analysis

We calculated the age standardised (WHO 2000–2025) incidence rate of lung cancer overall by smoking status.^16^

We stratified all models by recruitment sub-cohort (1991–1992, 1996–1997 and 2003–2007) to control for potential differences at the three recruitment waves. As previously described^15^ we used the Cox proportional hazards model, with age as the underlying time scale, to estimate crude and multivariate-adjusted hazard ratios (HRs) and 95% confidence intervals (CIs) for the associations between lung cancer and different measures of smoking exposure. Smoking exposure was defined using smoking status at cohort entry (never, passive, former, current or ever); for women who had ever smoked, further exposures were defined using smoking duration (1–9, 10–19, 20–29, ≥30 years), number of cigarettes smoked per day (1–9, 10–19, ≥20), number of pack-years of smoking (1–5, 6–15, >15) age at smoking initiation (≤ 17, >17–20, >20) years), and for former smokers: years since quit smoking (1–9, 10–20, > 20 years). The reference group is never active, never passive smokers throughout the manuscript, unless otherwise noted.

We included covariates that changed the HR estimate by at least 5% as confounders of the association between smoking and lung cancer.^17^ We included the following variables in the final multivariable models; age at enrolment, years of education (<10, 10–12, ≥13 years) and average alcohol consumption, in grams of alcohol per day (0, ≤4, 5–9, ≥10). We performed similar analyses without excluding participants with missing information on the covariates education and alcohol consumption. Indicator variables specific to missing in education and alcohol were used, after checking that these indicators were not statistically associated with risk of lung cancer. The other variables tested were BMI and physical activity, which did not change the estimates by at least 5%. Women who reported no alcohol consumption and those answering “seldom or never” had their alcohol consumption set to 0.

We tested for linear trends across categories of smoking exposure variables, excluding never smokers, by assigning a median score in order to account for the distance between categories, treating the variable as continuous in the analysis. We tested and found that the criteria for the proportional hazards assumption were met using Schoenfeld residuals (data not shown).

We calculated PAFs (%) to indicate what proportion of lung cancer cases could have been prevented in the population in absence of smoking. We used the formula PAF = *P*_e_ (RR_e_ − 1)÷ (*P*_e_ × RR_e_ + [1 − *P*_e_], where the notation *P*_e_ = the proportion of persons in the population exposed to the risk factor (i.e., ever smokers) and RR_e_ = the relative risk in the exposed compared with the unexposed group estimated through the HRs (ever compared with never active never passive smokers).^18^ We calculated the two-sided 95% CIs for the PAFs using the PUNAF Stata module.^19^

We performed analyses using STATA version 15.0 (Stata Corp.) and considered two-sided *P*-values ≤ 0.05 as statistically significant.

## Results

During the more than 2.3 million person-years of observation (mean follow-up time was 15.9 (±6.5) years, we ascertained 1507 lung cancer cases. At enrolment, the mean age of the participants was 48.9 (±8.3 s.d) years. Of these women, 69.3% (*n* = 98 795) reported to be ever (current 32.2%, former 37.1%) smokers, with 14.8 (± 12.9 s.d) years of smoking. Former smokers quit smoking 16.9 (±9.8 s.d) years ago. Among never smokers 17.6% (*n* = 25 082) reported to be passive smokers. Altogether 77.9% (*n* = 114 191) of the women reported to be alcohol drinkers, with a mean alcohol consumption of 3.9 (±6.1 s.d) grams per day. Mean years of education was 12.4 (±3.5) among the women. The age-standardised incidence rate for lung cancer among never, passive, and ever smokers was 13.4, 20.0, and 87.1 per 100,000 person-years respectively.

Table 1 shows that never and passive compared with current smokers, were more likely to have higher education, higher BMI and less likely to consume alcohol. Adenocarcinoma was the most common histological lung cancer type in both never and ever smokers. Squamous cell (15%)- and small cell (23%) carcinoma were found most commonly in current smokers. These two histological subtypes were almost absent in never smokers (Table 1).

**Table 1:** Selected characteristics of the study sample by smoking status, in the Norwegian Women and Cancer Study, 1991–2015, (*N* = 142 508).

Characteristics	Never	Passive^a^	Former	Current
Subjects	18 631 (13.1%)	25 082 (17.6%)	52 887 (37.1%)	45 908 (32.2%)
Person-years of follow-up	303 310	404 765	797 385	764 512
Age at enrolment, mean (±SD) Median [min-max]	51 (9) 51 [34–70]	48 (8) 48 [34–70]	50 (8) 50 [31–70]	47 (8) 47 [31–70]
Age at diagnosis, mean (±SD) Median [min-max]	66.6 (9.3) 67 [34–88]	63.0 (8.1) 64 [34–88]	63.3 (8.0) 64 [31–70]	63.0 (8.1) 63 [31–88]
Years since quit smoking, mean (±SD) Median [min-max]	NA^b^	NA^b^	16.6 (9.8) 15.5 [1.0–51.3]	NA^b^
Education years, mean (±SD) Median [min-max]	12.9 (3.8) 13 [0–45]	13.1 (3.6) 13 [0–43]	12.6 (3.6) 12 [0–40]	11.5 (3.1) 11 [0–40]
Physical activity (±SD) Median [min-max]	5.7 (1.8) 6 [1–10]	5.8 (1.9) 6 [1–10]	5.8 (1.9) 6 [1–10]	5.6 (2.0) 5 [1–10]
BMI^c^, mean (±SD) Median [min-max]	24.4 (3.9) 24 [15–64]	24.3(4.0) 24 [13–64]	24.6 (4.0) 24 [10–71]	23.6 (3.8) 23 [12–72]
Alcohol g/day, mean (±SD) Median [min-max]	2.2 (3.8) 0.8 [0–203]	3.1 (4.6) 1.7 [0–189]	4.4 (6.1) 2.4 [0–360]	4.4 (7.2) 2.1 [0–360]
Lung cancer overall	43	53	329	1,082
Adenocarcinoma^d^ (%)	26 (61)	34 (64)	173 (52)	459 (42)
Squamous cell carcinoma^d^ (%)	1 (2)	2 (4)	35 (11)	159 (15)
Small cell lung cancer^d^ (%)	2 (5)	2 (4)	45 (14)	246 (23)
Other nsclc^d,e^ (%)	12 (28)	13 (24)	59 (18)	138 (13)
Large cell carcinoma^d^ (%)	1 (2)	2 (4)	8 (2)	22 (2)
Excluded^d,f^ (%)	1 (2)	0 (0)	9 (3)	58 (5)

^a^Passive smokers include never smokers who lived with a smoker in their childhood and/or live with a smoker at enrolment of the study.

^b^NA (not applicable).

^c^Body mass index; weight in kilograms divided by the square of the heights in metres.

^d^The percentage of each histological subtype in each column is the percentage of the subtype of lung cancer overall in that column.

^e^Other nsclc; not specified non small cell lung carcinoma.

^f^Excluded; Other or not otherwise specified carcinoma.

Table 2 shows that when compared with never smokers, current smokers had a lung cancer hazard ratio that was almost 14-fold (HR 13.88, 95% CI 10.18–18.91) higher. For ever smokers there was a dose response for the different smoking exposures (smoking duration, cigarettes smoked per day and pack-years, all p-trend <0.001). The highest hazard ratios for lung cancer among ever compared with never smokers, was a 21 fold increase in risk of lung cancer among those who smoked > 20 cigarettes per day (HR 21.66, 95% CI 16.54–28.37) and for those who smoked > 15 pack-years (HR 21.24, 95% CI 15.52–29.06). Female never smokers exposed to passive smoking had a hazard ratio of 1.34 (95% CI, 0.89–2.01) compared with never smokers without any passive exposure.

**Table 2:** Crude^a^- and multivariate^b^ adjusted hazard ratio (HR) estimates for lung cancer with 95% confidence intervals (CIs) for different measures of smoking exposures, the Norwegian Women and Cancer Study, 1991–2015.

Smoking exposures	Cases (*n*)	HR^a^ (95% CI)	HR^b^ (95% CI)
Never^c^	43	1.00 (ref)	1.00 (ref)
Passive	53	1.33 (0.89–2.01)	1.34 (0.89–2.01)
Former	348	3.81 (2.77–5.23)	3.69 (2.68–5.09)
Current	1139	15.44 (11.37–20.98)	13.88 (10.18–18.91)
Ever	1464	7.67 (5.66–10.41)	6.80 (5.00–9.24)
*Smoking duration in ever smokers*^d^			
1–9	231	4.58 (3.29–6.39)	4.16 (2.98–5.82)
10–19	203	4.24 (3.05–5.91)	4.22 (3.02–5.90)
20–29	429	9.26 (6.75–12.71)	8.55 (6.20–11.77)
≥30	595	18.11 (13.25–24.74)	16.48 (12.02–22.60)
P-trend^e^		<0.001	<0.001
*Cigarettes smoked per day in ever smokers*^d^			
1–9	376	4.46 (3.57–5.58)	4.28 (3.42–5.37)
10–19	767	13.16 (10.65–16.28)	12.17 (9.81–15.10)
>20	125	23.07 (17.70–30.06)	21.66 (16.54–28.37)
P-trend^e^		<0.001	<0.001
*Pack-years in ever smokers*^d^			
1–5	199	4.01 (2.89–5.57)	3.81 (2.73–5.31)
6–15	429	8.40 (6.13–11.50)	7.90 (5.74–10.86)
>15	671	22.52 (16.53–30.69)	21.24 (15.52–29.06)
P-trend^e^		<0.001	<0.001
*Age at smoking initiation in ever smokers*^d^			
>20	334	6.33 (4.84–7.62)	5.76 (4.55–7.28)
17–20	739	9.95 (8.04–12.31)	9.08 (7.31–11.27)
≤17	315	6.07 (4.84–7.61)	5.70 (4.53–7.17)
P-trend^e^		<0.001	<0.001

^a^Adjusted for age.

^b^Adjusted for age, duration of education and alcohol consumption, all at enrolment.

^c^Never-active, never-passive smokers as reference group.

^d^The sum of cases in each interval in smoking duration, cigarettes day, pack-years and age at smoking initiation does not sum up to the total number of cases in ever smokers because of missing values.

^e^Test for trend excluding never smokers.

The overall results remained materially the same when we included participants with missing information on the covariates education and alcohol consumption (data not shown).

Table 3 shows that compared with current smokers, former smokers who had quit smoking 1–9 years ago had a 63% (HR 0.37, 95% CI 0.30–0.45) reduced risk, while those who had quit smoking >20 years ago had a 89% (HR 0,11, 95% CI 0.08–0.15) reduced risk of lung cancer (all p-trend < 0.001).

**Table 3:** Crude^a^- and multivariate^b^ adjusted hazard ratio (HR) estimates for lung cancer with 95% confidence intervals (CIs) for former smokers according to years since quit smoking, the Norwegian Women and Cancer Study, 1991–2015.

	Cases	HR^a^ (95% CI)	HR^b^ (95% CI)
Current smokers		1.00 (ref)	1.00 (ref)
1–9 years	90	0.34 (0.28–0.42)	0.37 (0.30–0.45)
10–19 years	56	0.16 (0.12–0.21)	0.17 (0.13–0.23)
>20 years	46	0.11 (0.08–0.14)	0.11 (0.08–0.15)
P-trend		<0.001	<0.001

^a^Adjusted for age.

^b^Adjusted for age, duration of education and alcohol consumption, all at enrolment.

The PAF of lung cancer was 85.3% (95% CI 80.0–89.2) for ever smoking.

## Discussion

In this nationally representative cohort of middle-aged Norwegian women, we found that never smokers exposed to passive smoking had a non-significantly higher risk of lung cancer compared with never smokers. More than eight in ten lung cancer cases are attributable to active smoking.

The proportion of 32.2% current smokers in our cohort is higher compared with current smokers among Norwegian women through our follow-up. The higher proportion of current smokers in our cohort could be a result of a higher proportion of smokers in the middle-aged women included in the study, compared with female smokers in the Norwegian population through our follow-up.^7^ In addition, there was a higher response in recruitment in the NOWAC study among women in northern Norway, which has the highest proportion of smokers in Norway.^20^

It is reassuring that although we have a relative small group of never smokers the age-standardised incidence rates for lung cancer that we found for never smokers not exposed to passive smoking, is in accordance with the 14.3 per 100 000 reported among never smokers not exposed to passive smoking in ‘The UK Million Women Cancer Study’.^21^

We found adenocarcinoma to be the most frequent histological subtype in both ever and never smokers, in accordance with other prospective cohort studies from the UK, the US and Norway.^21–23^

Our study confirms a consistent, strong dose-response relationship between active smokers and lung cancer risk, as has been previously shown in other cohort studies.^22,24^ We found an inverse dose response with time since quit smoking and risk of lung cancer among former smokers in accordance with prior findings.^22^ The risk of lung cancer in our study decreased almost to the level of a never smoker 20 years after quitting. Another cohort study, with 89 000 female participants of whom 144 former smokers were diagnosed with lung cancer, observed that the risk of lung cancer decreased to almost the level of a never smoker already 15 years after quit smoking.^24^

The magnitude of our estimate for lung cancer risk due to passive smoking was higher than those reported in two expert reports.^11,12^ The studies included in these reports ended their follow-up in the beginning of this century or before. Since the lag-time to develop lung cancer is several decades, the women in our study have had until 2015 to develop the disease, so this discrepancy is as expected. One limitation with our and other studies on passive smoking and risk of lung cancer is lack of power due to few cases among never smokers. A 2018 meta-analysis included a total of 1996 lung cancer cases from seven cohort studies, which reported between 11 and 136 lung cancer cases among passive smokers.^25^ Among the cohort studies included in the meta-analysis, the estimates for lung cancer risk among passive smokers was statistically significant in only one study which was from Korea, published in 1999. The Korean study included close to 160,000 female participants, and 79 lung cancer cases among women exposed to passive smoke from their spouse. They observed an increased risk of 90% among female passive smokers.^26^ Among the cohort studies in the meta-analysis, one of the studies were from Europe. The 2005 European study included in the meta-analysis comprised 95,947 women, among whom 70 were diagnosed with lung cancer; did not find any significantly increased risk of lung cancer in women exposed to passive smoke at home.^27^ One of the two studies from the US included in the meta-analysis, including 76,304 participants and follow-up until 2009, observed 152 lung cancer cases among never smokers. The US study found no association between exposure to passive smoking and lung cancer risk.^22^ Among the cohort studies included in the meta-analysis, only the study from Korea^26^ and one of the two studies from the US^22^ adjusted for occupation. In the ‘UK Million Women Study’, with follow-up through 2011, there were 1469 cases among the 634,039 never smokers, but exposure to passive smoking as a child and/or as adult was not associated with any significantly increased risk of lung cancer.^21^ This study did not adjust for occupation.

The PAF for ever smoking in our study corresponds to the 78% estimated for US white women. This study, published in 2014, had 173 lung cancer cases among the 5487 white women in the study.^28^ Similarly, the PAF value for ever smoking UK women, was estimated to be 80% in 2010. The PAF value in the UK women was based on indirect estimation from national vital statistics.^29^ To our knowledge, we are the first to report this high PAF for women based on individual data from a random nationwide sample. It shows that former and current smoking is causing more lung cancer among women than previously anticipated. We attribute this to the fact that we have follow-up until 2015. Furthermore, we expect that the PAF due to smoking among women may increase with a longer follow-up in future studies.

### Strengths and limitations of the study

The most important strength of our study is that it is a nationally representative cohort study allowing us to calculate the PAF of lung cancer due to smoking for women in their middle-age, based on individual data. From our previous studies^14,15,30,31^ we know that the smoking exposure and the cancer incidence^13^ reflect known smoking patterns^6^ and cancer incidence^32^ for Norwegian women. Thus, we are confident that our cohort is representative of the Norwegian female population, born between 1927 and 1965, both according to smoking exposure and outcome of our study. Other strengths are the high proportion of both current and former smokers, and a virtually complete follow-up through the National population-based registries. The lag period between smoking initiation, usually in adolescence and the end of follow-up resulted in a large number of cases, which gave us the ability to examine the dose response association and risk in both former and current smokers. Another strength is that we focus our PAF estimates on the comparison between ever versus never smokers which is the most interesting figure in a public health perspective. Also, only never smokers could possibly change smoking status during follow-up. Since very few Norwegian women start to smoke after age 30 and the mean age at enrolment of this study is more than 40 years, we are confident that the possible changes in smoking status among the never smokers during follow-up did not influence our PAF estimates.^33^ The limitations include lack of complete information on smoking status. We excluded nearly 11,000 women because of missing information due to passive smoking. We have few lung cancer cases among never smokers, resulting in a lack of statistical power. The prevalence of smoking among women in Norway continuously decreased during our follow-up (1991–2015). As we used baseline smoking exposure, our risk estimates and the PAF could be underestimated. Our current smokers at enrolment are during follow-up a mix of current and former smokers because some quit smoking. Therefore, our risk estimates for lung cancer among current smokers are actually not for current smokers. If no current smokers quit smoking during follow-up, our risk estimates would have been higher than we observed. Additionally, we lack information regarding smoking exposed occupations such as workers in bars and restaurants. Participants exposed to work related tobacco could have been classified as never smokers. This could reduce our risk estimates for passive and active smokers. Improvements for future studies include focus on the questionnaires regarding passive smoking and occupation, as well as smoking status.

As pointed out by Jha,^34^ the full effects of smoking can take up to 50 years to measure in individuals, and up to 100 years to measure in a population. We may therefore expect, that in Norway, where relatively many women have been smokers, our relatively high PAF may become even higher in future studies. Norway was also one of the first countries to introduce restrictive rules for tobacco control when ‘The Norwegian Tobacco Act’ entered into force in 1975.^35^ Milestones in ‘The Norwegian Tobacco Act’ are: Since 1988, there has been legal protection from exposure to tobacco smoke in workplaces, and since 2004, even a complete ban on smoking in bars and restaurants, and the legal age to buy tobacco was increased to 18 years in 1996. Our study shows that tobacco control campaigns and restrictions should continue to be a high priority in all countries with a high smoking prevalence. Further, smoking cessation programme among middle-aged women, should be given high priority. In summary, more than eight in ten lung cancer cases among middle-aged women in Norway could have been prevented if the women did not smoke.

## Data Availability

The dataset used during the current study is available from the corresponding author on reasonable request.
